# Sensorimotor adaptation of speech depends on the direction of auditory feedback alteration

**DOI:** 10.1121/10.0002876

**Published:** 2020-12-14

**Authors:** Hardik Kothare, Inez Raharjo, Vikram Ramanarayanan, Kamalini Ranasinghe, Benjamin Parrell, Keith Johnson, John F. Houde, Srikantan S. Nagarajan

**Affiliations:** 1 UC Berkeley - UCSF Graduate Program in Bioengineering, University of California San Francisco, San Francisco, California 94143, USA; 2 Educational Testing Service R&D, San Francisco, California 94105, USA; 3 Department of Neurology, University of California, San Francisco, San Francisco, California 94143, USA; 4 Department of Communication Sciences and Disorders, University of Wisconsin-Madison, Madison, Wisconsin 53715, USA; 5 Department of Linguistics, University of California, Berkeley, Berkeley, California 94720, USA; 6 Department of Otolaryngology—Head and Neck Surgery, University of California, San Francisco, San Francisco, California 94143, USA; 7 Department of Radiology and Biomedical Imaging, University of California, San Francisco, San Francisco, California 94143, USA

## Abstract

A hallmark feature of speech motor control is its ability to learn to anticipate and compensate for persistent feedback alterations, a process referred to as sensorimotor adaptation. Because this process involves adjusting articulation to counter the perceived effects of altering acoustic feedback, there are a number of factors that affect it, including the complex relationship between acoustics and articulation and non-uniformities of speech perception. As a consequence, sensorimotor adaptation is hypothesised to vary as a function of the direction of the applied auditory feedback alteration in vowel formant space. This hypothesis was tested in two experiments where auditory feedback was altered in real time, shifting the frequency values of the first and second formants (*F*1 and *F*2) of participants' speech. Shifts were designed on a subject-by-subject basis and sensorimotor adaptation was quantified with respect to the direction of applied shift, normalised for individual speakers. Adaptation was indeed found to depend on the direction of the applied shift in vowel formant space, independent of shift magnitude. These findings have implications for models of sensorimotor adaptation of speech.

## I. INTRODUCTION

The intent of human speech communication is to transmit information or express emotions and it relies on successful audition by the intended recipient. However, speech communication also involves continuous monitoring of auditory feedback by the speaker (Lee, 1950; Fairbanks, 1954; Houde *et al.*, 2002; Houde and Nagarajan, 2011; Guenther, 2016). The purpose of this monitoring may be to distinguish between self-generated and externally-generated speech (Korzyukov *et al.*, 2017; Subramaniam *et al.*, 2018) and also to detect and correct speech errors (Levelt, 1983, 1993).

Speech feedback monitoring has been examined in numerous experiments, showing that real-time changes in auditory feedback cause speakers to modify speech production in a compensatory manner. Speakers have been shown to compensate for changes in fundamental frequency (*F*0) (Burnett *et al.*, 1998; Jones and Munhall, 2000), vowel formant frequency (Houde and Jordan, 1998; Purcell and Munhall, 2006) and fricative centroid frequency (Shiller *et al.*, 2009). Persistent alterations to sensory feedback cause long-term changes in motor behaviour where sensory feedback alterations are anticipated. This learned compensatory process is called sensorimotor adaptation in speech and has been studied widely over the last couple of decades (Caudrelier and Rochet-Capellan, 2019).

Since sensorimotor adaptation involves adjusting articulation to counter the perceived effects of altering acoustic feedback, there are a number of factors that affect it. First, there is the complex relationship between acoustics and articulation (Stevens, 1968, 1998; Johnson, 2012; Gick *et al.*, 2013). In particular, we can consider the muscles, articulators, and motor programs involved in changes to vowel height and vowel backness that create acoustic changes in *F*1 and *F*2, respectively. Articulatory changes in vowel height and backness are achieved by relatively independent control of different tongue muscles (Takano and Honda, 2007; Gick *et al.*, 2013). For instance, the anterior genioglossus lowers and retracts the tongue tip and blade to produce low back vowels, the middle genioglossus can contract to lower the tongue body and pull it forward to produce low front vowels, whereas the posterior genioglossus can contract to pull the tongue root forward to produce high front vowels (Gick *et al.*, 2013). Therefore, the acoustic changes needed to counter different acoustic feedback alterations are likely accomplished by distinct articulatory motor programs that differ in their degrees of adaptability. In this way, adaptation response may depend on the changes in perceived vowel backness and vowel height created by the altered auditory feedback. Additionally, depending on the direction of the response to the altered auditory feedback, non-linearities in the acoustic-articulatory mapping (Stevens, 1989) would also create different changes in the magnitude of somatosensory and auditory feedback. This, in turn, would affect the magnitude of adaptation response since it is thought to be determined, in part, by the weighting of conflicting somatosensory and auditory feedback about the feedback error driving the adaptation (Lametti *et al.*, 2012). Second, factors like categorical perception create non-uniformities in speech perception (Pisoni and Remez, 2005). The acoustic change needed to counter feedback alteration may differ depending on how acoustic changes are perceived in the neighbourhood of the speech sound being produced. Indeed it has been shown that shifts nearer to vowel category boundaries cause enhanced feedback compensation responses (Niziolek and Guenther, 2013) and that individual differences in perceptual categories influence the magnitude of sensorimotor adaptation (Daliri and Dittman, 2019). The current study sought to test the following hypothesis: As measured by its magnitude and orientation, the extent to which the adaptation response opposes the applied feedback shift varies as a function of the direction of the applied feedback shift in vowel formant space.

## II. METHODS

Eighteen (ten female) participants were recruited for the study (average age = 28.83, standard deviation =11.82 years). Data on participants' linguistic background was not collected prior to the experiment but a survey on linguistic background was sent out to the participants after the experiments. Ten out of the 18 participants responded to this survey. Four respondents identified as trilingual or multilingual, three of them identified as bilingual, and the remaining three identified as monolingual English speakers. Six respondents indicated that they were most fluent in English and the remaining four stated that they were as fluent in English as another language. Six respondents said that English was the first language they were exposed to and all respondents said that English was their language of formal education. None of the participants reported any hearing loss or a history of speech and language deficits. The procedures of testing were explained to the participants and informed consent was obtained. The study was approved by the Committee on Human Research of the University of California, San Francisco.

Participants were seated in an audio booth (Eckoustic C-14A LP Mod. Rev., Eckel Industries of Canada, Morrisburg, Ontario, Canada) and were wearing a headset microphone (MicroMic C520, AKG Acoustics, Vienna, Austria). Baseline vowel formant frequency values for ten vowels (/ɛ/, /ɪ/, /i/, /e/, /æ/, /ɑ/, /o/, /ɔ/, /ʊ/, and /u/) were collected for all individuals using these prompt words, respectively: “bet,” “fit,” “meet,” the first part of the diphthong in “late,” “bad,” “car,” “hope,” “bought,” “book,” and “pool,” three samples for each vowel. Formants were tracked using linear predictive coding (LPC) and optimal LPC order for tracking was determined on a subject-by-subject basis (Schuerman *et al.*, 2017). Vowel formant frequency values for every subject were calculated by averaging values for every vowel across the three samples. These average values were used as the basis for determining the altered feedback in Secs. II A and II B.

Two experiments were conducted on separate days and are described in detail below. Fourteen of the 18 participants were able to participate in both experiments and the order of Experiments 1 and 2 was randomised for these subjects. The median difference between the date of Experiments 1 and 2 for these participants was six days. Two subjects could participate only in Experiment 1 and two could participate only in Experiment 2 due to scheduling conflicts.

During both experiments, participants wore circumaural headphones (DT 770 PRO 250 OHM, beyerdynamic, Heilbronn, Germany). Speech signals from the microphone were routed to a computer (Optiplex 9020 SFF, Dell, Round Rock, TX) with a Delta 44 sound card (M-Audio, Cumberland, RI) via a preamplifier (HR-MP2, Radio Design Labs, Prescott, AZ). These signals were analysed and re-synthesised by a real-time feedback alteration tool called feedback utility for speech processing (FUSP) designed by author J.F.H. This tool employs sinusoidal synthesis methods (Quatieri and McAulay, 1986) as described in previous studies (Katseff *et al.*, 2012).

### A. Experiment 1

**FIG. 1.**
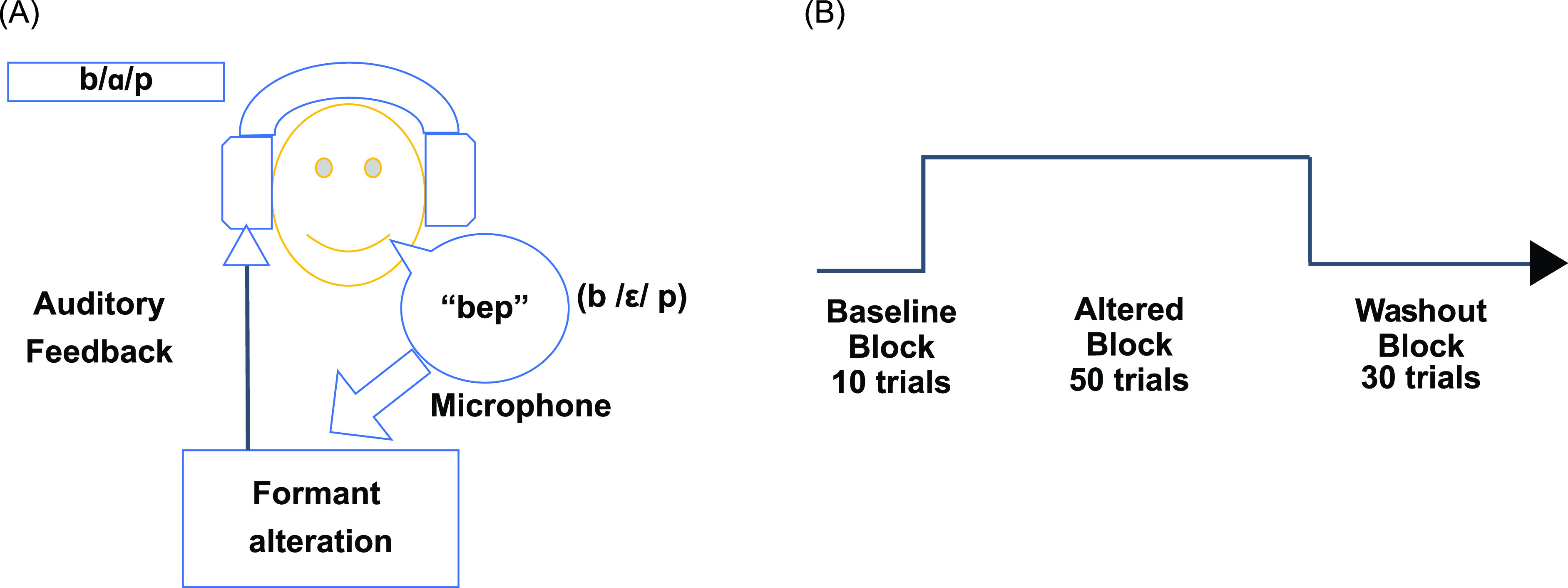
(Color online) (A) Schematic of the experimental setup. Participants spoke the syllable “bep” into a microphone when prompted; this input signal from the microphone was relayed to a digital signal processing unit which altered the first two formants (*F*1 and *F*2) during the altered block. This processed signal (altered or unaltered, depending on the block) was then played through headphones. (B) Schematic of the experimental design. Each case consisted of 90 trials which were divided into three blocks. The initial ten trials were part of the baseline block where the participants' feedback was unaltered, i.e., they heard what they said. This block was followed by the altered block of 50 trials where the formants from the speech signal were altered and fed back through the headphones. The last block was the washout block of 30 trials where formant alteration was turned off and feedback returned to normal.

This experiment consisted of six cases. Each case started with an unaltered block of ten trials, followed by an altered block of 50 trials, and concluded with an unaltered washout block of 30 trials. During every trial, subjects were prompted to read the nonsense word “bep” (/bɛp/). During the altered block, the first two formants were shifted from those of the subject's production of /ɛ/ to those of a different vowel sound, so that subjects heard formant frequency values corresponding to a perceivably-different vowel sound, depending on the case [see Figs. 1(A) and 1(B)]. The order of these six cases was randomised across participants. The shifts applied were based on individual baseline vowel formant frequency values collected prior to the experiment as described above. In particular, the shift in *F*1 was calculated as the difference between *F*1 of /ɛ/ and *F*1 of either /ɪ/ (as in fit), /i/ (as in meet), /e/ (as in the first part of the diphthong in late), /æ/ (as in bad), /ɑ/ (as in car), or /u/ (as in pool), depending on the case; the shift in *F*2 was calculated similarly. Thus, the magnitude and direction of shifts varied across cases and subjects (see Fig. 3).^1^

### B. Experiment 2

The sequence of blocks in this experiment was exactly the same as Experiment 1. The auditory feedback in the altered block was shifted towards the six target vowels from Experiment 1 but the magnitude of shift was just 50 Hz in all six cases. Again, the direction of applied shift was determined based on the baseline vowel formant frequency values for individual subjects collected prior to both experiments.

### C. Extraction of formant values

Each trial was visually inspected and played using custom-built speech analysis software called Wave Viewer written in matlab (The MathWorks, Inc., Natick, MA). This software analyses and displays the raw waveform as well as the spectrogram, formant tracks, time course of pitch, and amplitude of the speech signal. Trials were marked bad if formants were tracked incorrectly on the spectrogram or if subjects phonated multiple times within a single trial. All trials that were not marked as bad were considered good trials. For each good trial, formant tracks for *F*1 and *F*2 were extracted for the entire duration of vowel phonation, excluding the transitions to the flanking consonants, and mean *F*1 and *F*2 values were calculated. All good trials from the last 20 trials of the altered block were taken into consideration to determine adaptation response, as done in other sensorimotor adaptation studies (Jones and Munhall, 2000; Kitago *et al.*, 2013).

### D. Vocal tract length normalisation

According to the source-filter theory of speech production (Fant, 1960), the acoustic properties of speech are a function of the shape and length of the supralaryngeal vocal tract. To account for differences in participants' vocal tract lengths based on gender and age and to ensure that behavioural differences are not influenced by speaker differences, we performed the Δ*F* method of vocal tract length normalisation (Johnson, 2018). The ΔF value represents average spacing between vowel formants and is related to the talker's vocal tract length by the following formula:

$$\mathrm{Vocal}\;\mathrm{Tract}\;\mathrm{Length}=\frac{c}{2F},$$

where *c* = 35 000 cm/s or the speed of sound in warm, moist air.

Each speaker's Δ*F* value was calculated using the following formula:

$$\Delta F=\frac{1}{mn}\sum_{j}^{m}\sum_{i}^{n}\left[\frac{F_{ij}}{i-0.5}\right],$$

*where i = formant number and j = token number*.

For each participant, two formants (*F*1 and *F*2) and seven tokens (/ɛ/, /ɪ/, /i/, /e/, /æ/, /ɑ/, or /u/) were used to calculate Δ*F. F*1 and F2 values for every production in every trial and even those for the applied shifts were then vocal-tract-length-normalised by dividing them by Δ*F*.

**FIG. 2.**
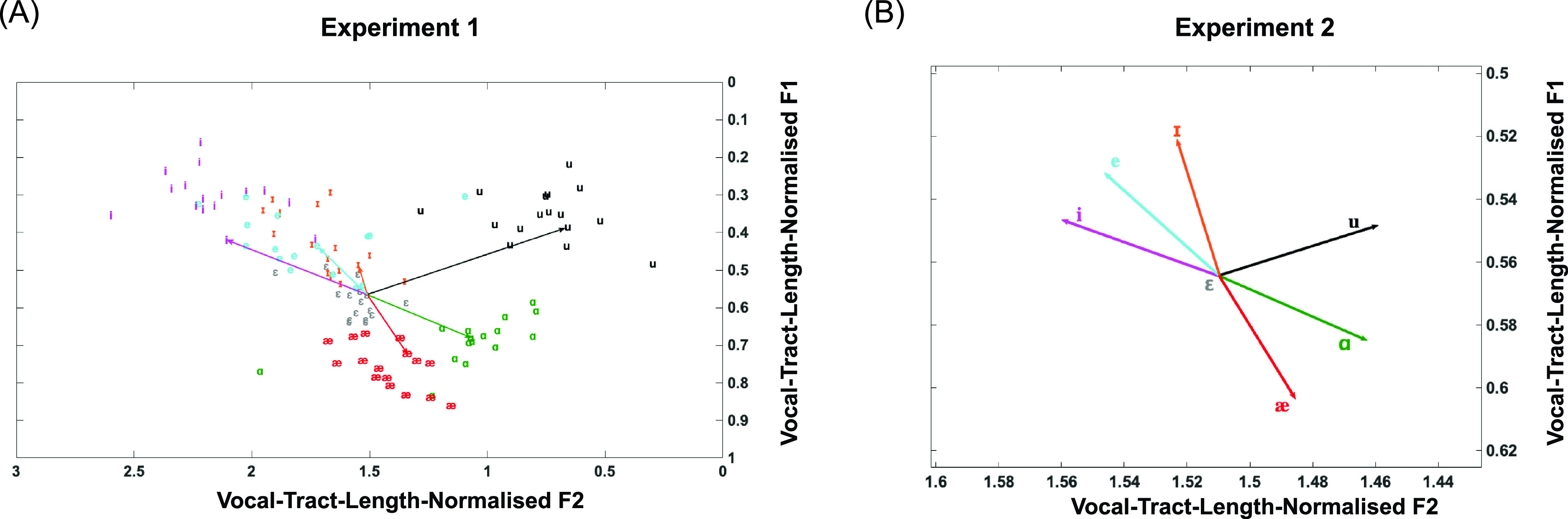
(Color online) (A) A scatter plot of the vowel locations of all 16 participants who took part in Experiment 1 represented in vocal-tract-length-normalised *F*1–*F*2 space. The *x* axis denotes vocal-tract-length-normalised *F*2 values and the *y* axis represents vocal-tract-length-normalised *F*1 values. The vectors represent six shifts for one particular participant. (B) A visual representation of the formant shifts for all six cases in Experiment 2 for the same example participant as in (A). The *x* axis denotes vocal-tract-length-normalised *F*2 values and the *y* axis represents vocal-tract-length-normalised *F*1 values.

**FIG. 3.**
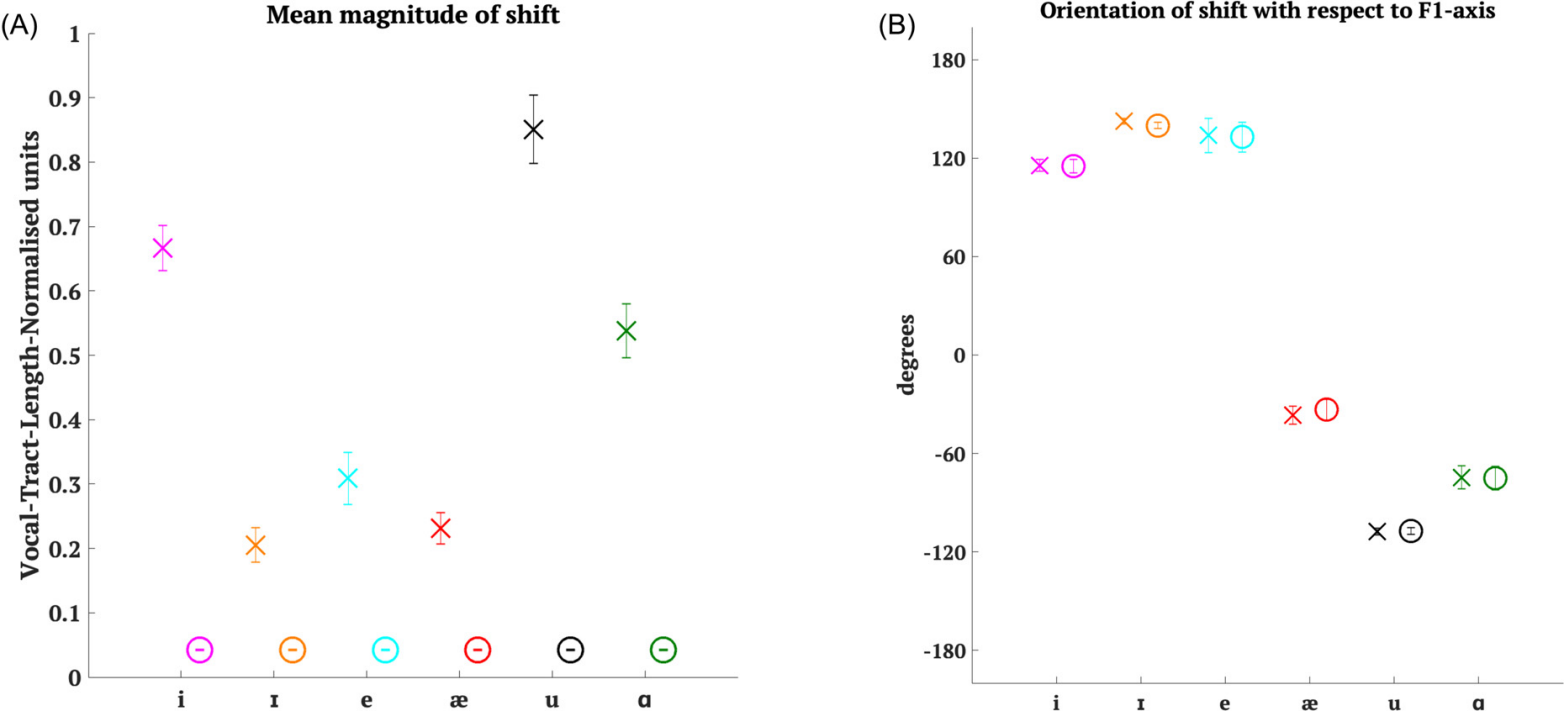
(Color online) (A) Mean magnitude of the applied shift vector (in vocal-tract-length-normalised units) for all six cases in Experiment 1 (crosses) and Experiment 2 (circles) averaged across participants. Error bars depict ± 1 standard error of mean. (B) Circular mean angle between the applied shift vector and the *F*1-axis (in degrees) for all six cases in Experiment 1 (crosses) and Experiment 2 (circles) averaged across participants. Positive values indicate that the angle is measured clockwise from a line parallel to the *F*1-axis to the shift vector [see Fig. 4(A)]. Negative values indicate that the angle is measured anticlockwise from a line parallel to the *F*1-axis to the shift vector. Error bars depict ± 1 standard error of mean.

Thus, vocal tract length normalisation converted *F*1–*F*2 space (Hz) into a normalised *F*1–*F*2 space for the purposes of the analysis. As an example, the shifts for one participant are shown in Fig. 2(A). Figure 2(B) shows the Experiment 2 shifts for the same participant as in Fig. 2(A). See also Fig. 3 for average shifts across participants.

### E. Vector resolution of response vector

**FIG. 4.**
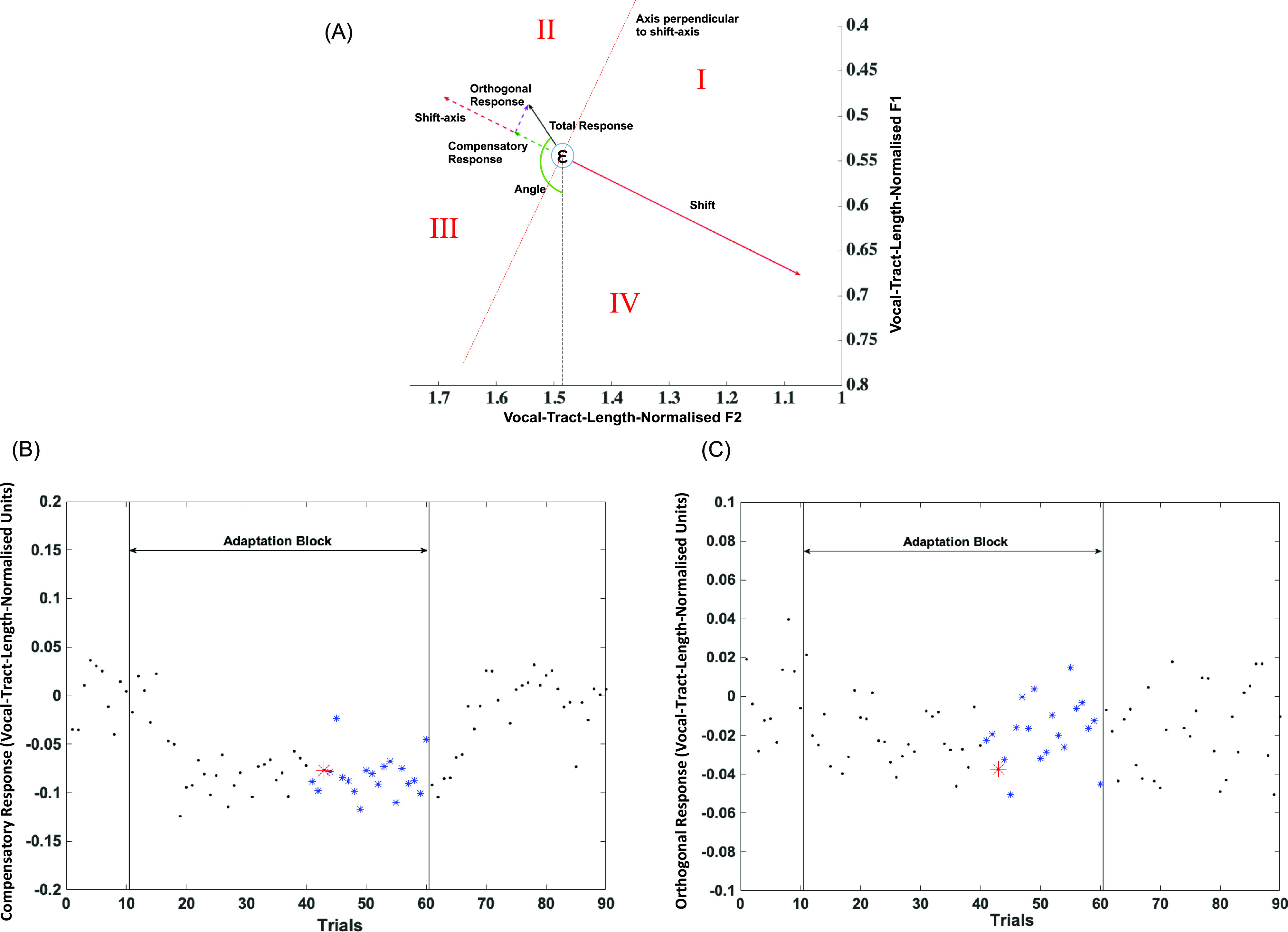
(Color online) (A) In this example, the CR is negative because it opposes the applied shift, the OR is negative as well because it lies in the second quadrant with respect to the direction of the shift. Quadrant numbers are mentioned in Roman numerals. Here, the TR takes the sign of the CR and is therefore negative. The angle between the response vector and a line parallel to the *F*1-axis was measured in degrees. (B) A scatter plot showing all 90 trials from one case for one participant on the *x* axis with CR (in vocal-tract-length-normalised units) represented by the *y* axis. The last 20 trials of the adaptation block, which were considered in the statistical analysis, are represented by asterisks (blue). The large asterisk (red) is the example trial shown in A. (C) A scatter plot showing all 90 trials from one case for one participant on the *x* axis with OR (in vocal-tract-length-normalised units) represented by the *y* axis. The last 20 trials of the adaptation block are represented by asterisks (blue). The large asterisk (red) is the example trial shown in (A).

To look at the results of Experiments 1 and 2, both the applied shift and response to this shift were expressed as vectors in the two-dimensional vocal-tract-length-normalised *F*1–*F*2 space. For every case in both experiments, subjects' baseline vowel formant frequency values for /ɛ/ were determined by averaging the frequency values for the first ten trials (baseline block). For every trial in every case, the total response (TR) was calculated as the length of the response vector in vocal-tract-length-normalised *F*1–*F*2 space from the point representing the baseline vowel formant frequency values to the formant frequency values of the vowel production in that particular trial. The response vector was resolved into a component along the axis of applied shift and a component perpendicular to the axis of applied shift [Fig. 4(A)], henceforth called compensatory response (CR) and orthogonal response (OR), respectively. CR was thus the scalar projection of the response on the shift axis. CR values were assigned a negative sign if the response was in the direction opposite to the applied shift and a positive sign if it was in the same direction [example in Fig. 4(B), see also Fig. 4(A)]. OR values were assigned a negative sign if the response vector was located in the first or second quadrant of the cartesian coordinate system with the shift-axis as the reference and a positive sign if the response vector was located in the third or fourth quadrant [example in Fig. 4(C), see also Fig. 4(A)]. TR took the same sign as that assigned to CR. The angle between the response vector and the *F*1-axis (ANG) was also measured for every trial.

We suggest that each of these four measures defines a particular aspect of sensorimotor adaptation. TR is a direct measure of the articulatory realisation and thus represents the size of the response. CR is a measure of the actual compensation in articulation to “nullify” the effect of the applied shift. OR represents the component of the response that does not contribute to compensation but contributes to the total size of the response. Collectively, CR and OR measure the efficiency of the response. ANG indicates the invariant direction of response, not with respect to the applied shift but in two-dimensional vocal-tract-length-normalised space and represents the acoustic and articulatory consequences of the adaptive response.

### F. Statistical analyses

In this subsection, we describe the statistical analyses we ran. We first talk about the tests we ran to test the dependence of TR, CR, and OR on the independent variables in both experiments and across experiments. We then talk about how we dealt with circular quantities in our statistical analyses. We proceed to talk about the dependence of ANG on the independent variables in both experiments and across experiments. Last, we talk about how we controlled for false positives due to multiple significance testing.

#### 1. TR, CR, and OR in Experiments 1 and 2

In both experiments, the primary independent variable of interest was the direction of applied auditory feedback (angle of shift in vowel formant space). To test the hypothesis that the adaptation response depended on the direction of applied shift, we evaluated separate linear mixed effect models (LMM) for three of the dependent variables TR, CR, and OR (implemented in sas 9.4 (sas Institute Inc., Cary, NC). For each model and for data from both experiments, the magnitude of shift and gender were included as covariates in the LMM. The LMM provides a principled framework for examination of the effect of an independent variable of interest on a dependent variable of interest even in the presence of covariates that are correlated with the independent variable of interest. For Experiment 2, the applied shifts were uniform in magnitude in *F*1–*F*2 space across participants and cases (50 Hz). However, after vocal-tract-length-normalisation, the shift values were uniform only within-participant and not across participants. Therefore, the magnitude of applied shift was also included as a covariate for Experiment 2 (even though the magnitude difference across participants was minimal).

Across all the above LMMs, subjects were treated as a random effect. For each of these LMMs, we compared a fixed effects model without varying slopes to a model with varying slopes and we were unable to reject the null hypothesis of equal slopes using the type III *F*-statistic for an interaction term (Littell *et al.*, 2006). Therefore, we chose to report the models with random intercepts.

To evaluate the secondary hypotheses that adaptation responses depended on the relative backness or height of the applied shift, we ran separate and reduced LMMs for vowel backness and height. For relative vowel height, we grouped shifts towards vowels into two categories based on the relative height with respect to the produced vowel /ɛ/ (relatively higher: /i/, /ɪ/, /e/, and /u/; relatively lower: /æ/ and /ɑ/). For relative vowel backness, we grouped shifts into two groups according to relative vowel backness with respect to the produced vowel /ɛ/ (relatively to the front: /i/, /ɪ/, and /e/; relatively to the back: /æ/, /u/, and /ɑ/). For these reduced LMMs, across both experiments, the dependent variables were TR, CR, and OR.

#### 2. Comparison of TR, CR, and OR across the two experiments

To examine differences in response measures across the two experiments, we used data from the 14 participants who took part in both experiments. To account for differences in the magnitude of applied shift across the two experiments, normalised measures were computed and used. Normalised compensation ratio (NCR), normalised orthogonal ratio (NOR), and normalised total ratio (NTR) were calculated for every subject and case by first averaging CR, TR, and OR values across the last 20 trials of the adaptation block and dividing these averaged values by the magnitude of the applied shift. First, separate LMMs were computed with experiment number as an independent categorical variable, and NTR, NCR, and NOR as the dependent variables. The angle of applied shift as an independent variable of interest and an interaction term (experiment number by angle of shift) were also included in these models. Second, separate and reduced LMMs were examined for the effect of relative vowel height and backness. For these reduced models, as before, shifts were grouped by either relative vowel height or backness. These reduced LMMs included experiment number and vowel height or backness as the independent variables of interest, and also included interaction terms. For these LMMs, subjects were treated as a random effect in all models and the models included random slopes and intercepts (Littell *et al.*, 2006).

#### 3. Dealing with circular quantities

The primary independent variable of interest (angle of shift) and one dependent variable (ANG) were circular quantities or values that were measured along a circle. Due to the periodic nature of such quantities, they may require statistical analyses designed for circular data (Mardia and Jupp, 2009; Cremers and Klugkist, 2018).

##### a. Circular independent variable of interest (angle of shift).

To account for the independent variable, angle of shift, being a circular quantity, we used target vowel direction (/ɪ/, /i/, /e/, /æ/, /ɑ/, or /u/), a categorical variable, as a surrogate for angle of shift to represent the direction of applied shift. We reran all the LMMs involving angle of shift as the primary variable of interest for TR, CR, and OR in both experiments as well as the normalised measures comparing values from Experiments 1 and 2. We did not find evidence suggesting that replacing the circular quantity with a surrogate variable changes the findings of our study. Therefore, we chose to report the results from the LMMs with angle of shift as an independent variable in the subsequent sections because angle of shift captures the between-subject variability in the direction of applied shift (because angle of shift is a continuous variable).

##### b. Circular dependent variable (ANG).

To account for the dependent variable, ANG, being a circular quantity, we first verified that our data followed the von Mises distribution (von Mises, 1918) using Watson's *U*^2^ goodness-of-fit test (Watson, 1961; Lockhart and Stephens, 1985) (Experiment 1: *U*^2^-statistic = 2.8248, *p* < 0.01, *R*-squared = 0.2255; Experiment 2: *U*^2^-statistic = 1.9357, *p* < 0.01, *R*-squared = 0.2243; Experiment 1 versus Experiment 2: *U*^2^-statistic = 4.3611, *p* < 0.01, *R*-squared = 0.2208). For each experiment, we then ran three separate parametric Watson-Williams (WW) tests as one-way analysis of variance (ANOVA) tests for circular data (Watson and Williams, 1956; Berens, 2009; Cremers and Klugkist, 2018) with ANG as the dependent variable and target vowel direction, target vowel relative height and target vowel relative backness as the categorical independent variables in those three tests respectively. For the comparison of response measures across both experiments, we ran three separate parametric Harrison-Kanji (HK) tests as a two-way ANOVA for circular data (Harrison and Kanji, 1988; Berens, 2009) with ANG as the dependent variable. The two independent categorical variables for the three tests were, respectively: experiment number and target vowel direction, experiment number and target vowel height, experiment number, and target vowel backness. An interaction term between the independent variables was also included for each HK test.

#### 4. Multiple correction

Finally, to control for false positives due to multiple significance testing, *p*-values for significance were adjusted using the Benjamini-Hochberg false discovery rate (*α* = 0.05) procedure (Benjamini and Hochberg, 1995), and only findings that survived this adjusted significance threshold were reported.

## III. RESULTS

### A. Experiment 1

**FIG. 5.**
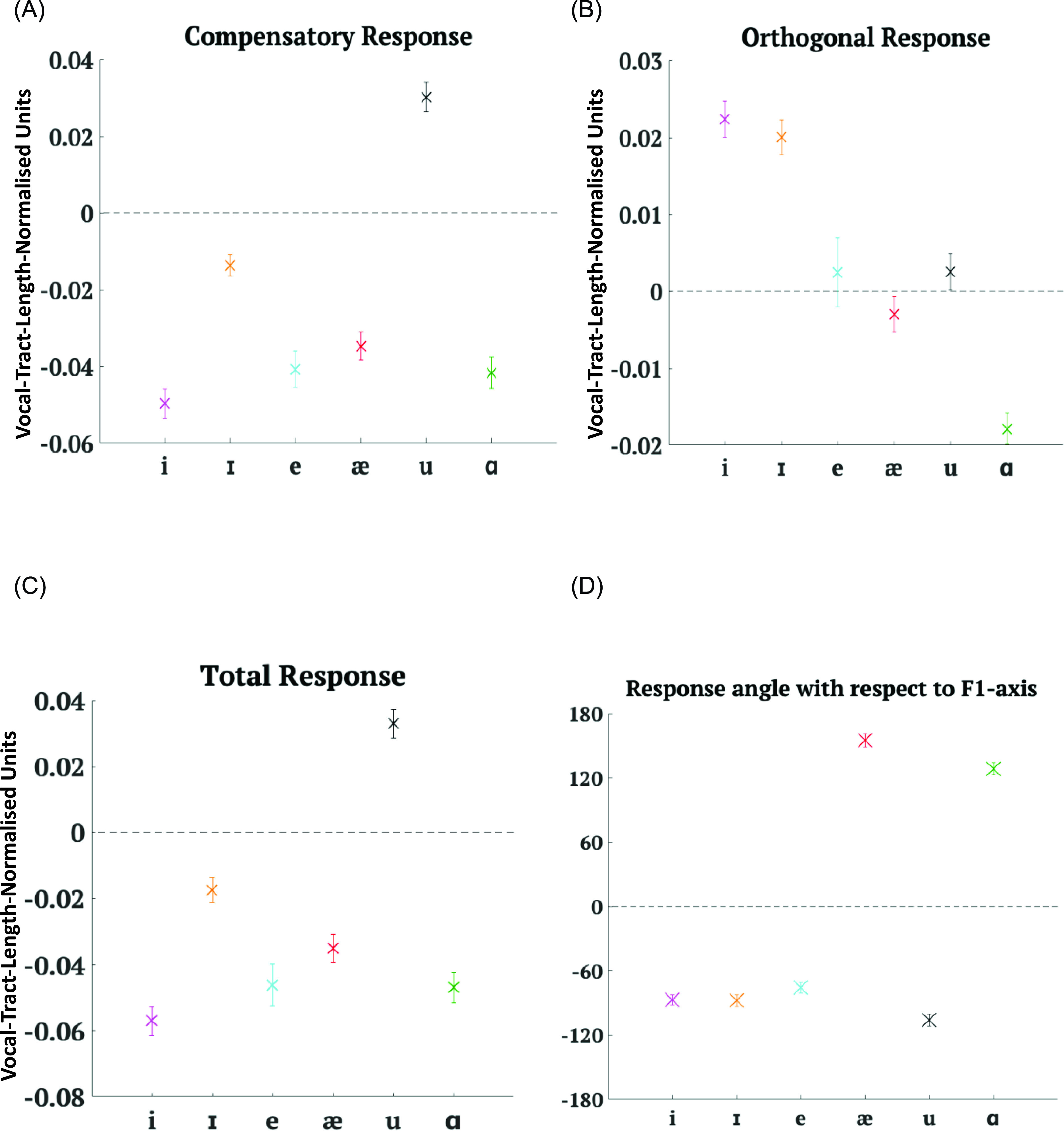
(Color online) Mean results for all six cases in Experiment 1 averaged across participants. Error bars depict ± 1 standard error of mean. (A) Mean CR (in vocal-tract-length-normalised units). Negative values indicate a response opposing the applied shift, whereas positive values indicate a response following the applied shift. (B) Mean OR magnitude (in vocal-tract-length-normalised units). Negative values indicate a response in the first and second quadrant with the direction of the shift vector as the reference. Positive values indicate a response in the third or fourth quadrant. (C) Mean TR (in vocal-tract-length-normalised units). Negative values indicate a response opposing the applied shift, whereas positive values indicate a response following the applied shift. (D) Circular mean angle between the response vector and the *F*1-axis (in degrees). Negative values indicate that the angle is measured clockwise from the *F*1-axis to the response vector. Positive values indicate that the angle is measured anticlockwise from the *F*1-axis to the response vector.

Results showed [Fig. 5(A)^1^ that the CR was in the direction opposite to the applied shift in all cases except the shift towards /u/. Although most CR values indicated that participants tried to oppose (or compensate for) the shift whether the angle of shift was positive or negative, the value of CR varied along with the angle of shift [*F*(1,1828) = 39.97, *p* < 0.0001]. If we look at CR values for shifts towards /i/ and /ɪ/, although the angles of shift were close to each other and on the same side with respect to the *F*1-axis [Fig. 3(B)], their CR values were very different from each other. CR also varied along with the magnitude of shift [F(1,1828) = 119.51, p < 0.0001]. The largest shifts, on average, were towards /u/ [Fig. 3(A)] and produced a following response. The shifts towards /ɪ/ and /æ/ were smaller than the rest but still produced differing CR values.

If we examine the OR [Fig. 5(B)], we observe that shifts towards /e/, /æ/, and /u/ produced a small OR but shifts towards /i/, /ɪ/, and /ɑ/ engendered a comparatively larger OR. On average, with the notable exception of /u/, the pattern seems to match that of angle of shift, with positive angles of shift producing a positive OR and negative angles of shift producing a negative OR. Indeed, OR was dependent on angle of shift [*F*(1,1828) = 87.97, *p* < 0.0001]. Looking at the figure, there seems to be a natural grouping based on whether the shifts were towards vowels that are relatively higher (a decrease in *F*1 values) than /ɛ/ or whether the shifts were towards vowels that are relatively lower (an increase in *F*1 values). OR, however, is not dependent on magnitude of shift.

In Fig. 5(C), we see that TR shows a pattern that is very similar to CR. It was dependent on angle of shift [*F*(1,1828) = 44.27, *p* < 0.0001] and magnitude of shift [*F*(1,1828) = 93.48, *p* < 0.0001]. As expected, responses to shifts towards /u/ stood out as an exception because of their following nature.

When we look at ANG values in Fig. 5(D), we again observe clear groupings in responses to shifts towards vowels that are relatively higher than /ɛ/ versus shifts towards vowels that are relatively lower than /ɛ/. Because shifts towards /i/, /ɪ/, and /e/ caused responses that were compensatory in nature and their angles of shift with respect to the *F*1-axis were positive, it makes sense to see that their ANG values were negative. Similarly, since shifts towards /ɑ/ and /æ/ also produced CRs and their angles of shift were negative, their ANG values were positive. It is then obvious that /u/ with its following response would have an average ANG value that is negative. Thus, ANG, the angle made by the response vector with the *F*1-axis or the orientation of the response in formant frequency space regardless of the applied shift, depended on the target vowel direction [*F*(5,1845) = 154.06, *p* < 0.0001].

OR was dependent on whether the shift was towards higher vowels or lower vowels [*F*(1,1829) = 91.28, *p* < 0.0001] and so was ANG [*F*(1,1845) = 737.24, *p* < 0.0001]. Even CR [*F*(1,1829) = 34.47, *p* < 0.0001] and TR [*F*(1,1829) = 22.27, *p* < 0.0001] showed dependency on relative vowel height. CR and TR values for shifts towards /æ/ and /ɑ/, on average, did not differ much from those for shifts towards /i/, /ɪ/, and /e/. However, the differences found could be explained by the exceptional following behaviour in the case of shifts towards /u/.

Also, CR [*F*(1,1829) = 37, *p* < 0.0001], TR [*F*(1,1829) = 38.65, *p* < 0.0001], OR [*F*(1,1829) = 91.49, *p* < 0.0001] and ANG [*F*(1,1845) = 474.83, *p* < 0.0001] were all dependent on whether shifts were towards vowels to the front of /ɛ/ versus to the back of /ɛ/. Responses to shifts towards /u/ may be responsible for the effect in CR and TR, whereas in OR it could be the relatively large negative value for shifts towards /ɑ/.

To summarise, in Experiment 1, all response measures, CR, OR, TR, and the angle between the response vector and *F*1-axis (ANG) depend on the angle of applied shift. Additionally, CR and TR also depend on the magnitude of shift. All four measures were also dependent on the relative vowel height and vowel backness of the target vowel of the altered auditory feedback.

### B. Experiment 2

**FIG. 6.**
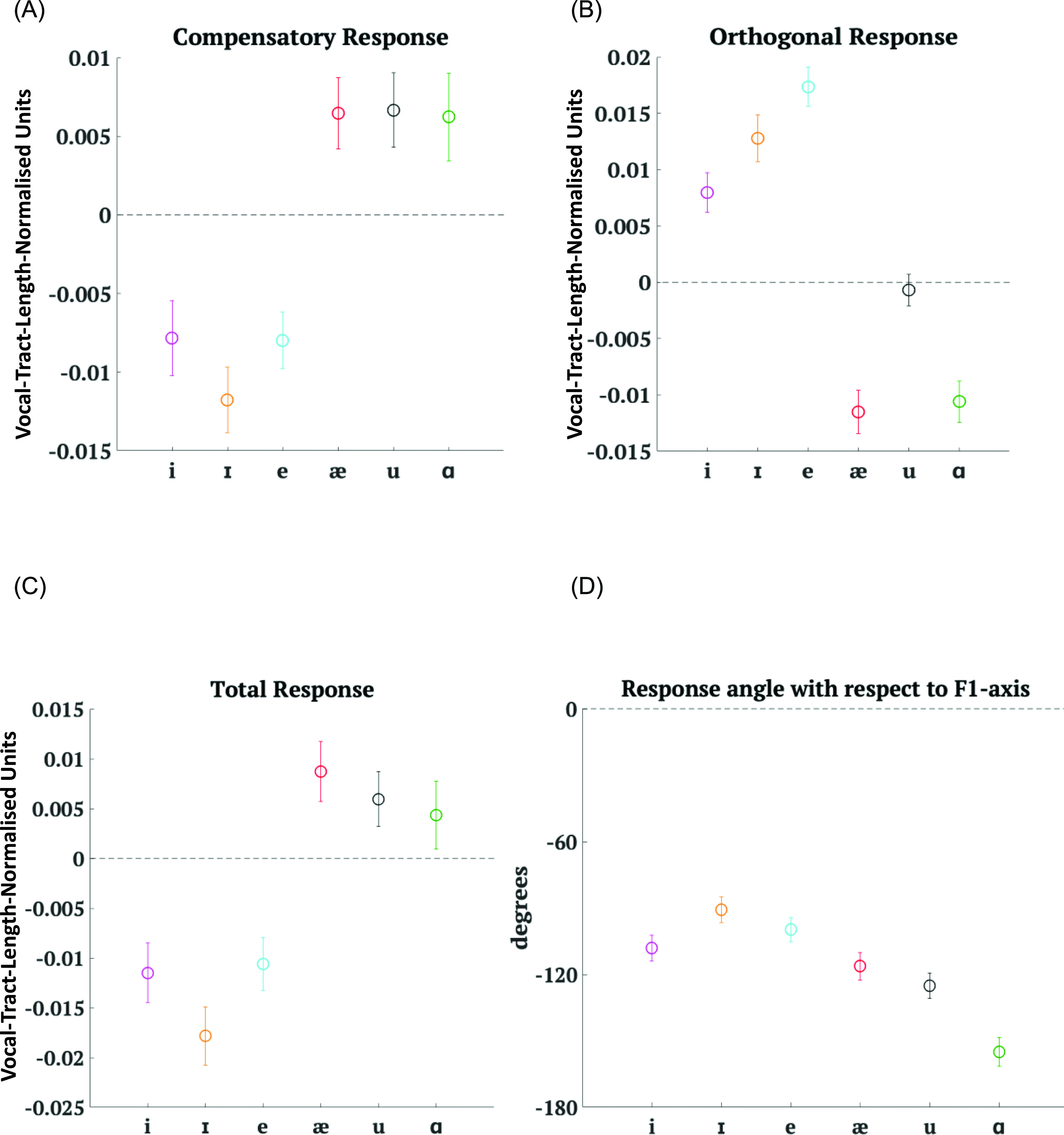
(Color online) Mean results for all six cases in Experiment 2 averaged across participants. Error bars depict ± 1 standard error of mean. (A) Mean CR (in vocal-tract-length-normalised units). Note: The *y* axis scale differs from the *y* axis scale in Fig. 5(A). (B) Mean OR magnitude (in vocal-tract-length-normalised units). Note: The *y* axis scale differs from the *y* axis scale in Fig. 5(B). (C) Mean TR (in vocal-tract-length-normalised units). Note: The *y* axis scale differs from the *y* axis scale in Fig. 5(C). (D) Circular mean angle between the response vector and the *F*1-axis (in degrees). Note: The *y* axis scale differs from the *y* axis scale in Fig. 5(D).

Results from Experiment 2 showed^1^ that the smaller shifts produced two clear groups of CR values [Fig. 6(A)]. For shifts in the directions of /i/, /ɪ/, and /e/, the responses were, on average, compensatory in nature, whereas responses to shifts towards /æ/, /u/, and /ɑ/ were, on average, following in nature. There was a clear subdivision based on shifts towards vowels that are relatively to the front of /ɛ/ (increase in *F*2) versus shifts towards vowels that are relatively to the back of /ɛ/ (decrease in *F*2). For angles of shift that were positive, participants seemed to compensate, whereas they seemed to follow when angles of shift were negative. Thus, CR depended on angle of shift [*F*(1,1823) = 75.55, *p* < 0.0001]. CR also depended on magnitude of shift [*F*(1,1823) = 7.05, *p* = 0.008].

In Fig. 6(B), we can observe that although the average OR value for shifts towards /u/ was small just like in Experiment 1, the average value in Experiment 2 was negative. While OR values for shifts towards /u/ were an exception in Experiment 1, OR values in Experiment 2 were positive for angles of shift that were positive with respect to the *F*1-axis (i.e., shifts with an increase in *F*2 values) and negative for angles of shift that were negative (shifts with a decrease in *F*2 values). There was a clear divide along shifts towards vowels that are relatively to the front of /ɛ/ and shifts towards vowels that are relatively to the back of /ɛ/. Thus, OR was dependent on angle of shift [*F*(1,1823) = 101.75, *p* < 0.0001]. OR was not dependent on magnitude of shift.

TR values [Fig. 6(C)] showed a pattern similar to CR. TR was dependent on angle of shift [*F*(1,1823) = 76.98, *p* < 0.0001] with positive angles of shift causing a net CR and negative angles of shift causing a net following response. TR was dependent on magnitude of shift [*F*(1,1823) = 4.13, *p* = 0.0423].

In Fig. 6(D), ANG values showed an interesting pattern in Experiment 2. All shifts produced ANG values that were negative on average. This makes sense when you note that positive angles of shift caused a CR and thus should have negative ANG values. Similarly, since negative angles of shift caused a following response, one would expect to see negative ANG values for these shifts. ANG values varied according to the target vowel direction [*F*(5,1840) = 12.3, *p* < 0.0001].

In Experiment 2, although the main divide was not along the lines of vowel height, TR [*F*(1,1824) = 32.14, *p* < 0.0001], CR [*F*(1,1824) = 31.44, *p* < 0.0001], OR [*F*(1,1824) = 171.79, *p* < 0.0001] and ANG [*F*(1,1840) = 33.53, *p* < 0.0001] all depended on whether the shifts were towards higher vowels or towards lower vowels as compared to /ɛ/. The larger effect size in the case of OR as compared to CR and TR was perhaps a result of the average OR value of shifts towards /u/ being closer to the average OR values of the other relatively higher vowels.

The response measures that showed a clear dichotomy between shifts towards vowels that are relatively to the front of /ɛ/ and shifts towards vowels that are relatively to the back of /ɛ/ [Figs. 6(A)–6(C)] were CR [*F*(1,1824) = 71, *p* < 0.0001], OR [*F*(1,1824) = 196.28, *p* < 0.0001] and TR[*F*(1,1824) = 67.01, *p* < 0.0001]. Even ANG [*F*(1,1840) = 37.72, *p* < 0.0001] depended on relative vowel backness.

To summarise, in Experiment 2 as well, all response measures, CR, OR, TR, and the angle between the response vector and *F*1-axis (ANG), depended on angle of shift in Experiment 2 just as in Experiment 1. Additionally, CR and TR also depended on variations in the magnitude of applied shift. All four measures also depended on whether the applied shift involved an increase or decrease in *F*1 value and whether they involved an increase or decrease in *F*2 value.

### C. Accounting for perceptual differences in magnitude of applied shift

The applied shifts in our experiments were in Hertz values. In Experiment 2, the shift was 50 Hz for all cases. Vocal tract length normalisation of the vowel formant space maintained shifts of equal magnitudes across directions of shift for a given subject but the magnitudes varied slightly across subjects, as mentioned before in Sec. II F 1.

However, shifts designed in Hertz values, although uniform in *F*1–*F*2 vowel space, may not be uniform on a psychoacoustic or perceptual scale. Each subject's individual perceptual scale for formant frequency values may be too idiosyncratic to be captured on a fixed scale like the mel scale (Greenwood, 1997). Nevertheless, to account for the possibility of perceptual differences for different shifts in both experiments, we converted all shift magnitudes to mels^1^ (O'Shaughnessy, 1987) and reran the statistical models including magnitude of shift in mels as a covariate instead of magnitude of shift in vocal-tract-length-normalised units. We did not observe any differences in the fixed effect of angle of shift in either experiment. It is important to note that this conversion to mels would cause shift magnitudes to vary both within and across subjects.

### D. Comparison of response measures across experiments

**FIG. 7.**
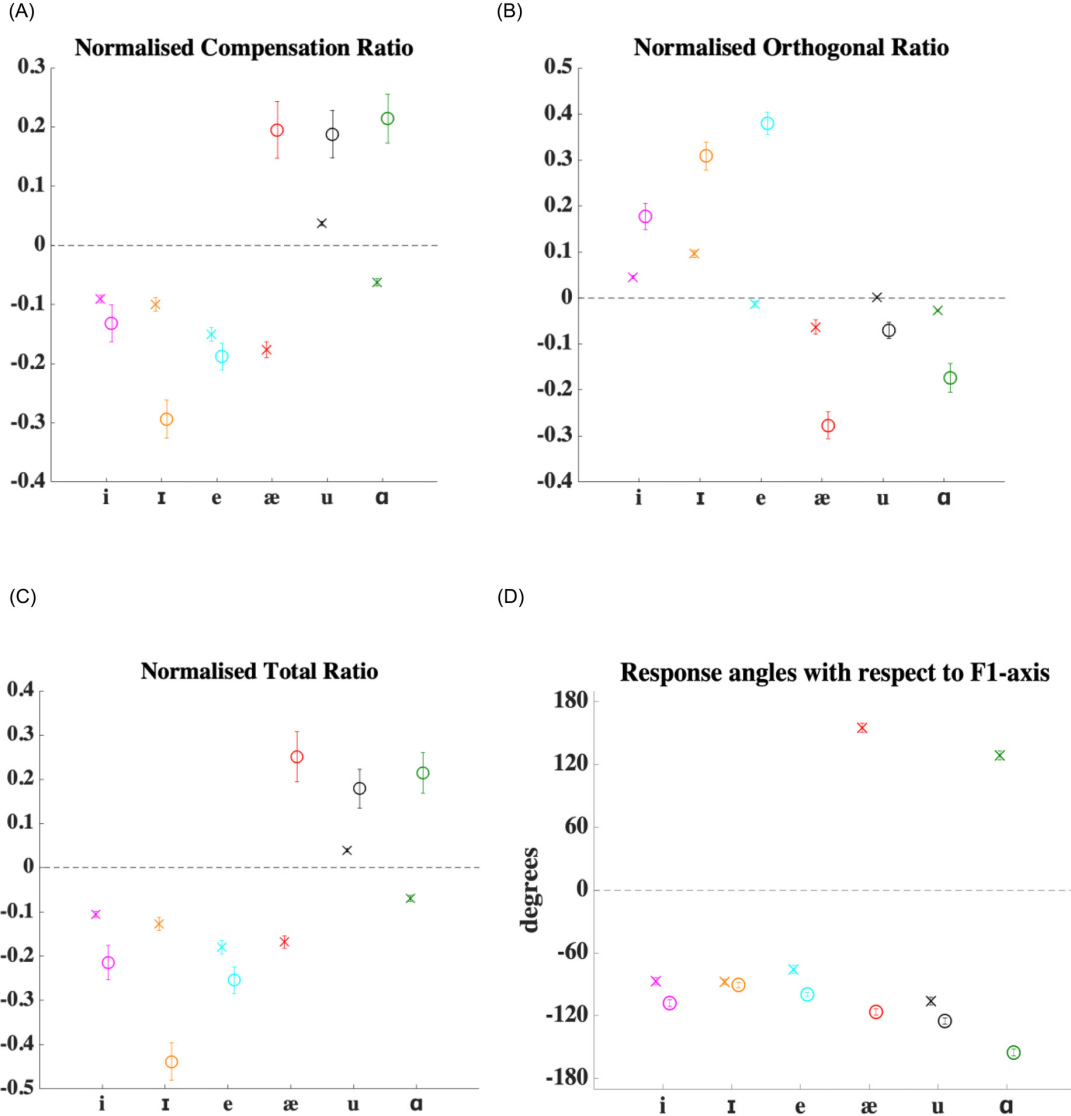
(Color online) Mean results for 14 of the 18 participants who took part in both Experiments 1 and 2. Error bars depict ± 1 standard error of mean. (A) Average CRs for both experiments were normalised (i.e., divided by the applied shift magnitude) and were called NCRs. the NCR values for Experiments 1 (crosses) and 2 (circles) are shown. (B) Average ORs for both experiments were normalised (i.e., divided by the applied shift magnitude) and were called NORs. The NOR values for Experiments 1 (crosses) and 2 (circles) are shown. (C) Average TRs responses for both experiments were normalised (i.e., divided by the applied shift magnitude) and were called NTRs. The NTR values for Experiments 1 (crosses) and 2 (circles) are shown. (D) Circular mean angle between the response vector and the *F*1-axis (in degrees) for Experiments 1 (crosses) and 2 (circles) are shown.

In Fig. 7(A), it can be seen that for all shifts, the magnitude of normalised compensatory response (NCR), i.e., the CR divided by the magnitude of applied shift, was larger in Experiment 2 than in Experiment 1. In proportion to the applied shift magnitude, participants had larger CR values in Experiment 2 as compared to Experiment 1. Therefore, NCR was dependent on experiment number [*F*(1,3225) = 30.25, *p* < 0.0001], angle of shift [*F*(1,3225) = 119.9, *p* < 0.0001] and there was a significant interaction between experiment number and angle of shift [*F*(1,3225) = 28.89, *p* < 0.0001]. Since NCR values were normalised CR values and since CR was dependent on angle of shift in both experiments, it comes as no surprise that NCR would depend on angle of shift. Moreover, the significant interaction term indicates that the pattern of covariation of NCR with angle of shift depended on experiment number. NCR depended on target vowel backness [*F*(1,3225) = 106.01, *p* < 0.0001]. This dichotomy between shifts towards vowels to the front of /ɛ/ and vowels to the back of /ɛ/ is clearer in Experiment 2 than in Experiment 1 seen in the interaction term between experiment number and target vowel backness [*F*(1,3225) = 34.59, *p* < 0.0001]. Similarly, NCR also depended on target vowel height [*F*(1,3225) = 35.29, *p* < 0.0001] and its interaction with experiment number [*F*(1,3225) = 33.28, *p* < 0.0001].

Figure 7(B) tells us that normalised orthogonal response (NOR) values too were larger in Experiment 2 than in Experiment 1. Smaller shifts in Experiment 2 produced proportionally larger OR values. However, NOR was not found to be dependent on experiment number [*F*(1,3225) = 1.9, *p* = 0.1678]. Looking at the pattern of NOR across cases, it seems that the mean NOR values for each experiment were not that different from each other. NOR was dependent on angle of shift [*F*(1,3225) = 102.39, *p* < 0.0001] and there was a significant interaction between experiment number and angle of shift [*F*(1,3225) = 42.21, *p* < 0.0001]. This reiterates that the angle of shift determined OR values and how OR covaried with angle of shift depended on experiment number. There was also a significant target vowel backness effect for NOR [*F*(1,3225) = 188.07, *p* < 0.0001] and this effect had an interaction with experiment number [*F*(1,3225) = 100.41, *p* < 0.0001]. The shifts towards front versus shifts towards back divide was clearly much more evident in Experiment 2. NOR depended on the target vowel height [*F*(1,3225) = 145.79, *p* < 0.0001] and its interaction with experiment number [*F*(1,3225) = 69.34, *p* < 0.0001].

Normalised total response (NTR) [Fig. 7(C)] showed a pattern that was similar to NCR. TR was proportionally larger for the smaller shifts in Experiment 2 as compared to Experiment 1. Although NCR was dependent on experiment number and NOR was not, NTR was indeed dependent on experiment number [*F*(1,3225) = 14.51, *p* = 0.0001]. NTR also varied according to angle of shift [*F*(1,3225) = 125.27, *p* < 0.0001] and there was a significant interaction between experiment number and angle of shift [*F*(1,3225) = 36.35, *p* < 0.0001]. Similar to the effects seen in NCR and NOR, NTR was also dependent on the target vowel backness [*F*(1,3225) = 106.58, *p* < 0.0001] and its interaction with experiment number [*F*(1,3225) = 34.65, *p* < 0.0001]. Shifts towards vowels to the front of /ɛ/ produced a consistent oppositional response in Experiment 2, whereas shifts toward vowels to the back of /ɛ/ consistently produced following responses. NTR also depended on the target vowel height [*F*(1,3225) = 42.18, *p* < 0.0001] and its interaction with experiment number [*F*(1,3225) = 32.45, *p* < 0.0001].

The angle between the response vector and *F*1-axis (ANG) values could not be normalised by magnitude of shift values but on comparison, the pattern in Experiment 1 was different from that in Experiment 2, as observed before. However, statistically from the HK test, we did not find that ANG was dependent on experiment number [*χ*^2^ = 2.7231, *p* = 0.2563]. When ANG values from both experiments were taken into consideration, they were still dependent on target vowel direction [*χ*^2^ = 243.1855, *p* < 0.0001]. The interaction term between experiment number and target vowel direction was also significant [*χ*^2^ = 149.5644, *p* < 0.0001], indicating that the way ANG values covaried with the angle of shift depended on the experiment number.

Like the other measures of response, ANG was also dependent on target vowel backness [*χ*^2^ = 183.5941, *p* < 0.0001] and target vowel height [*χ*^2^ = 197.9663, *p* < 0.0001] and their interactions with experiment number respectively [*χ*^2^ = 65.8612, *p* < 0.0001 and *χ*^2^ = 137.1501, *p* < 0.0001].

**FIG. 8.**
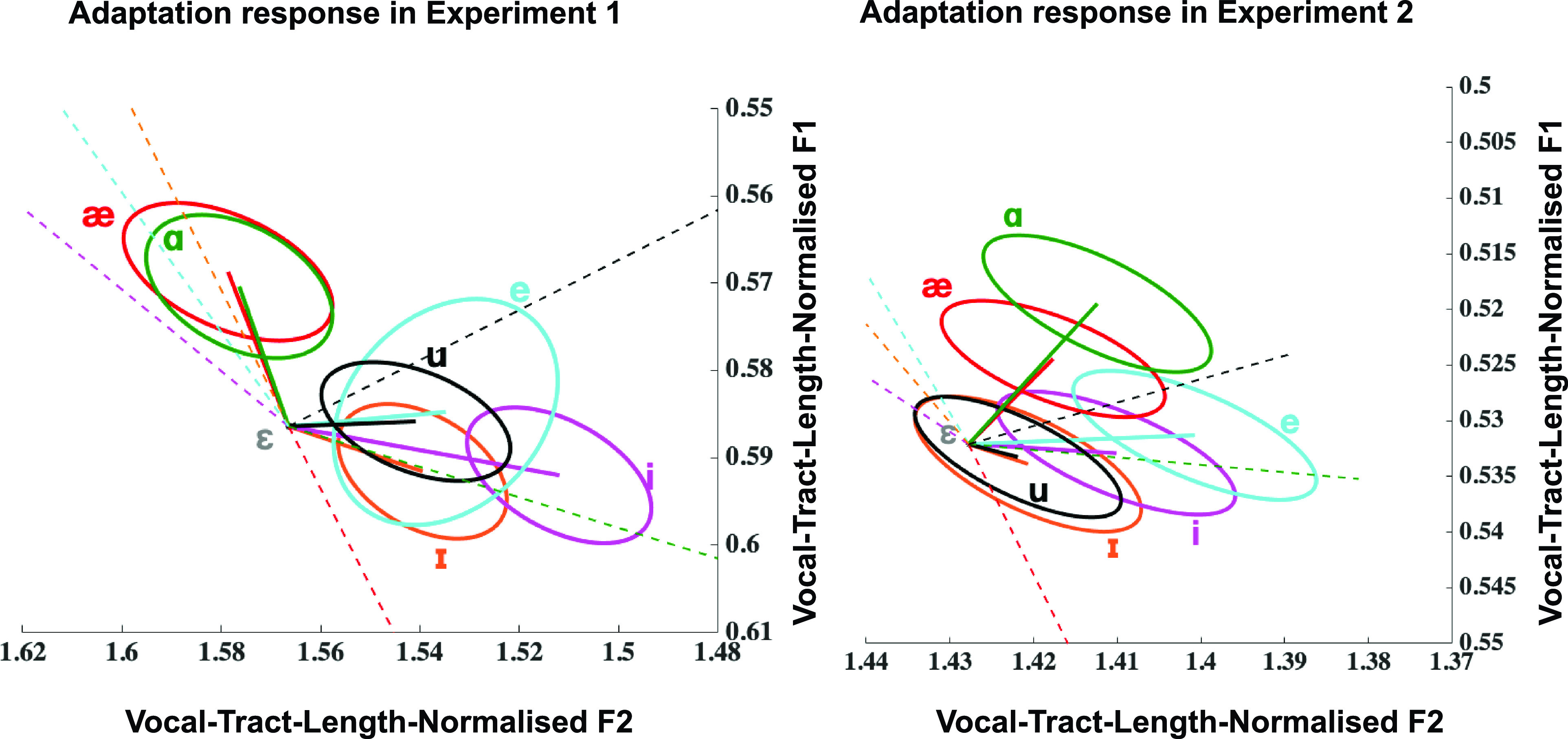
(Color online) (A) and (B). Mean baseline vocal-tract-length-normalised ɛ formant frequency values for 14 of the 18 participants who took part in both Experiments 1 and 2 and standard error ellipses representing adaptation responses to the shifts applied (shifts represented by dashed lines). Note: The representation of both the applied shifts and the corresponding responses shown here is based on vector averages across both participants and trials. However, actual applied shift vectors were participant-specific and adaptation responses were calculated for each subject relative to these participant-specific shift vectors. For each experiment, the average shift and response vectors were computed relative to the corresponding baseline vocal-tract-length-normalised ɛ formant frequency values for that experiment.

Further details about the responses in both experiments can be seen upon examination of the two-dimensional plot of produced formant changes (solid lines with standard error ellipses) in response to applied shift vectors (dashed lines) [Fig. 8(A) for Experiment 1 and Fig. 8(B) for Experiment 2]. The representation of both the applied shifts and the corresponding responses shown here is based on vector averages across both participants and trials (note, however, actual applied shift vectors were participant-specific and adaptation responses were calculated for each subject relative to these participant-specific shift vectors). It can indeed be seen from Fig. 8(A) that in Experiment 1, responses to shifts towards /i/, /ɪ/, /e/, /æ/, and /ɑ/ were compensatory in nature, whereas responses to shifts towards /u/ were following in nature. In Fig. 8(B), we see that in Experiment 2, responses to shifts towards /i/, /ɪ/, and /e/ remain compensatory in nature, whereas responses to shifts towards /æ/, /u/, and /ɑ/ were following in nature. The figure highlights the qualitative similarities and differences between the two experiments. In both experiments, the responses to shifts towards /u/ are following and shifts towards /i/ and /ɪ/ show a CR. The only major qualitative differences between responses in Experiments 1 and 2 are for the shifts towards vowels that are lower and to the back of /ɛ/, namely, /æ/ and /ɑ/, which change from compensatory in Experiment 1 to following in Experiment 2.

## IV. DISCUSSION

In this study, we conducted two experiments to investigate the possibility that sensorimotor formant adaptation varies as a function of the direction of formant shift. Both experiments looked at responses to altered formant feedback in different directions from the same vowel. In Experiment 1, the shifts were from /ɛ/ to six other vowels. In Experiment 2, the shifts were in the same six directions as in Experiment 1 but of equal magnitudes in Hertz values. For both experiments, we quantified shift magnitudes and responses in *F*1–*F*2 space that was normalised for vocal tract length (Johnson, 2018). We comprehensively characterised the vector describing the participants' formant adaptation responses and found that formant adaptation indeed depends on the direction of the applied shift. Across both experiments, response characteristics also depended on relative height and backness of the vowel target to which feedback was altered. These results and their implications for models of speech motor control are discussed below.

### A. Formant adaptation depends on direction and magnitude

Results from Experiment 1 showed that responses were dependent on the direction of the applied shift independent of differences in applied shift magnitude across the six different vowel targets. For five of these six shifts, the response was compensatory in nature with /u/ being the only exception. All response measures depended on the direction of applied shift in vocal-tract-length-normalised *F*1–*F*2 space. To further examine the specificity of the effect of shift direction on adaptation responses, in Experiment 2, for each subject, the applied shifts were in the same six directions as in Experiment 1 but of equal magnitudes in formant frequency space. Results again showed that adaptation responses depended on the direction of shift. For both experiments, the main effect of angle of shift on TR, CR, and OR held true even after controlling for differences in magnitudes of applied shift in perceptual units.

There were differences in adaptation response observed across the two experiments. Responses for smaller shifts of Experiment 2 were not merely a scaled version of responses to larger shifts in Experiment 1. In particular, responses to shifts towards /æ/ and /ɑ/, which were compensatory in Experiment 1, were following in Experiment 2.

Together, these findings suggest that the simple model of CRs opposing the applied feedback shift is not accurate. In fact, the pattern of CRs is quite a complex function of direction and magnitude of the applied shift.

### B. Possible explanations for direction dependence of adaptation

Sensorimotor adaptation in speech is a response to persistent feedback alteration. It can be described as learning to change articulation to compensate for a perceived error between predicted and actual sensory feedback, i.e., a sensory feedback prediction error. Sensorimotor adaptation in the production of vowels has previously been shown to depend on the size of the formant alteration (Katseff *et al.*, 2012) and the vowel produced when experiencing the alteration in single-word production in laboratory settings (Mitsuya *et al.*, 2015) or natural connected speech (Lametti *et al.*, 2018). Mitsuya *et al.* (2015) showed that adaptation to *F*1 shifts depended on whether the *F*1 shift was an increase or a decrease, providing preliminary evidence that adaptation may vary as a function of shift direction. Results from the current study suggest that adaptation also depends on the direction of alteration in vowel formant space. Models of speech production currently do not account for these results. To begin to explore how these models could be modified, we consider various contributory factors that could potentially explain the direction dependence of adaptation observed in this study.

One possible explanation for why adaptation differs as a function of direction in vowel formant space involves considering the combination of two factors, acoustic to articulatory non-linearities inherent in speech production (Stevens, 1989) and the hypothesis that sensorimotor adaptation is a balance between compensating for sensory prediction errors in audition and somaesthesis (Katseff *et al.*, 2012; Lametti *et al.*, 2012). The amount of articulatory change needed to counter formant feedback perturbation varies as a function of the direction of that perturbation in vowel formant space. This variation in articulatory response arises from the non-uniformity of the relationship between articulation and acoustic consequences. In particular, the quantal theory of speech production (Stevens, 1989) states that changes of articulatory configuration during speaking cause changes to the resulting acoustic output in a non-monotonic manner, i.e., in some parts of the vocal tract, a small difference in articulatory positioning corresponds to a large acoustic difference, whereas in other parts, even a large difference in articulatory positioning is not sufficient to cause a large change in the acoustic consequences. Because of this, different degrees of articulatory change are needed to counter acoustic perturbations in different directions. These differing articulatory changes would cause speakers to experience varying levels of somatosensory feedback change. In particular, CRs to shifts towards higher vowels may differ from CRs to shifts towards lower vowels because of changes in expected somatosensory feedback through the lateral contacts of the tongue at the palate.

The articulatory change that corrects for an auditory feedback prediction error will in turn generate a somatosensory feedback prediction error in the opposite direction. The resulting trade-off between correcting for auditory feedback prediction errors and competing somatosensory feedback prediction errors may partly account for the observed direction dependence of adaptation.

A second factor contributing to direction dependence could arise from articulatory constraints on producibility of CRs, i.e., the ability to reconfigure the human vocal tract to a state that achieves compensation. There are physical limits to the dimensions of the vocal tract and CRs may not be possible for all directions of applied shifts. Moreover, the required CRs may involve moving the vocal tract into regions where the speaker has limited phonetic experience based on their language.

A related interesting finding from the current study is that responses to formant shifts also depended on the height and backness of the vowel target to which feedback was altered, relative to the intended vowel production. Changes to vowel height and backness are achieved by moving different parts of the tongue. Changes towards back vowels may require changes in the tongue body, whereas changes towards front vowels may require changes to the tongue blade (Stevens, 1998). Therefore, it is natural that the compensatory adjustments would group according to whether the compensatory change is one of fronting or backing. Similarly, because different muscles are involved in tongue raising or tongue lowering (Gick *et al.*, 2013), compensatory adjustments would also group according to whether the compensatory change is one of lowering or raising.

The observed effects of vowel height and backness are consistent with the two factors describing tongue position as described by Harshman *et al.* (1977) that can be explained by tongue biomechanics (Perrier *et al.*, 2007). Furthermore, as stated before, CRs to shifts towards higher vowels may differ from CRs to shifts towards lower vowels because of corresponding changes in expected somatosensory feedback through the lateral contacts of the tongue at the palate. Greater palatal contact would be more consistent with auditory feedback conveying the percept of higher vowels and so the absence of this palatal contact may have an effect on the adaptation response to shifts towards higher vowels.

A possible third factor contributing to the observed direction dependence of adaptation is categorical-like perception of vowels (Kuhl, 1991). We know that behavioural and cortical responses to formant feedback alterations depend on whether the applied shifts cross a vowel category boundary (Niziolek and Guenther, 2013). In the current study, the shifts for Experiment 1 were carefully chosen for each participant such that the resulting feedback crossed vowel category boundaries and was a perceivably-different vowel sound. Shifts in different directions would cross different numbers of category boundaries, thus affecting the perceptual salience of the applied shifts. This would result in directional dependence of adaptation responses. However, in Experiment 2, although we did not measure categorical boundaries in our study, it is likely that all the shifts stayed within the categorical boundary of /ɛ/ for three reasons. First, Niziolek and Guenther (2013) shifted formant feedback from /ɛ/ such that it did or did not cross the categorical boundary, which they measured perceptually, to either /æ/ or /ɪ/. The average shift applied in their study for crossing the categorical boundaries was 122.2 mels. Second, we observe from baseline formant measurements of the production of different vowels in our study that formant distances in *F*1–*F*2 space from /ɛ/ to the two closest vowels, /æ/ and /ɪ/, were ∼165 mels. Even if we assume that the category boundary is at the midpoint between these vowels, shifts of ∼83 mels would be required to cross categorical boundaries. Third, the natural variability of the productions of the vowel /ɛ/ across participants was found to be ∼30 mels (two times the standard deviation of the mean baseline /ɛ/ production = 29.91 mels), which is likely to be well within the vowel category of /ɛ/. Nevertheless, in spite of all shifts being within the same category, we observed direction dependence of the adaptation response. Therefore, taken together, the results of Experiments 1 and 2 suggest that direction of applied shift is by itself a determining factor for the adaptation response. There is some evidence that sensorimotor adaptation is sensitive to the ease with which the altered vowel feedback can be assimilated into a known phonological category (Mitsuya *et al.*, 2013). Adaptation is also sensitive to the lexical status of the altered vowel feedback sound (Bourguignon *et al.*, 2014). While we did not focus on lexical and phonological categories in our experiments, it would be an important research path to pursue in the future.

The manipulations in Experiment 1 were focused on distinctiveness of the shifted auditory feedback to a perceivably-different vowel sound and how one responds to these feedback alterations. Although the prompt word for both experiments was the same (“bep”), the formant frequency values of the shifts were determined prior to the experiments in a separate pre-test session where participants produced words containing different vowels. These words had different consonantal environments. Rhotacisation, diphthongization, and other coarticulatory contexts encountered in the production of these words would affect vowel formant frequencies and therefore the formant shifts used in our experiments. However, since the consonantal environment was fixed in the actual experiments we do not think these factors affect the adaptation responses which were always measured relative to the applied shift. We also note that the durational characteristics of a particular vowel may covary with its formant frequency values. The feedback may be perceived as unnatural because of differences in vowel length between the natural production of a particular vowel and its altered-feedback version. It is true that our feedback shifts did not impact the duration of the perceived vowel. Five of the six feedback shifts in Experiment 1 would be considered shifts from a short vowel to a long vowel (with the only exception being /ɪ/, where the shift would be from a short vowel to another short vowel; Rositzke, 1939). Nevertheless, this may not be an important factor affecting our findings for the following reasons. First, vowel duration has small or relatively modest effects on vowel identity (Hillenbrand *et al.*, 2000). Furthermore, in American English (the language of our study participants) the distinction between short and long vowels is not so sharp as in other dialects of English like British English (Wells, 1962). Second, vowel durations are sensitive to the consonant context (House and Fairbanks, 1953). In particular, a well-known phenomenon called pre-fortis clipping applies to our experiments because the coda consonant in our prompt was a fortis obstruent (/p/). It has been shown that this consonant context can reduce long vowel durations by almost 40%–50% (House, 1961; Kluender *et al.*, 1988; Wells, 1990). Therefore, having the vowel formants shifted to long vowel identities while retaining the short vowel duration of the produced vowel /ε/ will not sound unnatural due to perceptual expectations of pre-fortis clipping of long vowel durations before the coda consonant /p/.

### C. The curious case of following responses

There were a number of following responses observed in our experiments. Here we discuss the possible reasons for such responses.

Participants, on average, tended to follow the shift from /ɛ/ towards /u/ in Experiment 1. There could be two reasons why this may have occurred: (1) /u/ was the only rounded vowel amongst the six shifts and may provide rich somatosensory feedback during rounding. An altered auditory feedback sounding like /u/ without the presence of lip-rounding somaesthesis may drive the participants towards “rounding” their production by producing something like /u/. We only tracked the first two formants in the current study but this line of investigation could be explored in future experiments by looking at the effect of feedback manipulations on participants' *F*3 values. (2) Producibility of CRs depends on articulatory constraints, also mentioned in the previous section. Responses for the shift towards /u/ in Experiment 1 may be following in nature because an opposing response to the shift would require an articulatory configuration that would produce a formant pattern lying outside the vowel space for most people, i.e., an articulatory configuration that has never been achieved by the participant during speaking. In a study investigating compensation strategies for labial perturbation of the rounded vowel [u] (Savariaux *et al.*, 1995), the authors suggest that complete acoustic compensation may be impossible due to speaker-dependent articulatory constraints. They further suggest that these constraints are due more to speaker-specific internal representation of articulatory-to-acoustic relationships rather than to any anatomical or neurophysiological limitations. In our study, these constraints on production may manifest themselves as a following response.

In Experiment 2, following responses were observed for shifts towards /u/, /æ/, and /ɑ/. Previous studies have suggested that large feedback shifts can be interpreted as targets rather than production errors (Burnett *et al.*, 1998; Behroozmand *et al.*, 2012) causing a following response. Here, we suggest that this following phenomenon can also be observed for smaller shifts.

### D. Implications for models of speech production

Our results have implications for current models of sensorimotor behaviour in speech production (Houde and Nagarajan, 2011; Tourville and Guenther, 2011; Parrell *et al.*, 2019). While in theory, these models include influences beyond auditory feedback that control responses to auditory feedback perturbations, these models have not adequately elaborated in detail the effects of extra-auditory influences like somaesthesis and articulatory constraints on determining auditory feedback responses. Incorporating the effects of these extra-auditory influences in models of speech motor control would provide a quantitative framework to assess whether these factors alone or their combination can lead to the pattern of direction dependence of adaptation we found in this study.

### E. Limitations

It is important to acknowledge the limitations of our study. First, although the participants were all English-speakers, they were recruited from the San Francisco Bay Area, where people come from diverse linguistic backgrounds (Hall-Lew, 2010) and may be exposed to varying degrees of acoustic and articulatory goals during everyday speech. Although we used shifts tailored to each subject's vowel space in this study, how these findings vary across different linguistic groups needs to be further explored. Second, the shifts that we used do not cover the whole gamut of an individual's vowel space. We had to limit the scope of our study, due to constraints like experimental time, to six shifts that we felt were a good representation of back vowels, front vowels, closed vowels, and open vowels. While there's a general relation between vowel height-backness and *F*1–*F*2 (Stevens and House, 1955; Fant, 1960), the actual relationship is more complex (Stevens, 1998) and future studies should look at articulatory measures along with acoustic measures. Third, our focus was on the first two formants that were shifted and analysed in the current experimental design. In order to look at phenomena like lip-rounding in rounded vowels where *F*3 is implicated (Stevens and House, 1955), we would track and shift *F*3 in such experiments. Last, we did not test our participants for differences in vowel discriminability and vowel category boundaries. The shifts applied in Experiment 2 were equal in absolute frequency values but may not be equal on a psychoacoustic scale that takes perceptual differences into account. Future versions of this experiment could include perceptual testing and perceptually-equal shifts.

## Footnotes

^1^ See supplementary material at https://www.scitation.org/doi/suppl/10.1121/10.0002876 for figures with individual participants' mean shift magnitude and angle of shift values; for figures with all data points from Experiments 1 and 2; for figures with shift magnitudes in mels.
